# Application of Tarkhineh Fermented Product to Produce Potato Chips With Strong Probiotic Properties, High Shelf-Life, and Desirable Sensory Characteristics

**DOI:** 10.3389/fmicb.2021.657579

**Published:** 2021-05-17

**Authors:** Amir Kiani, Yousef Nami, Shahab Hedayati, Daniel Elieh Ali Komi, Farjam Goudarzi, Babak Haghshenas

**Affiliations:** ^1^Regenerative Medicine Research Center (RMRC), Kermanshah University of Medical Sciences, Kermanshah, Iran; ^2^Department of Food Biotechnology, Branch for Northwest & West Region, Agricultural Biotechnology Research Institute of Iran, Agricultural Research, Education and Extension Organization (AREEO), Tabriz, Iran; ^3^Students Research Committee, Kermanshah University of Medical Sciences, Kermanshah, Iran; ^4^Cellular and Molecular Research Center, Cellular and Molecular Medicine Institute, Urmia University of Medical Sciences, Urmia, Iran

**Keywords:** probiotic, Tarkhineh, potato chips, storage stability, sensory properties

## Abstract

The application of Tarkhineh texture to protect probiotics in potato chips has been investigated as the main goal in this paper. In this study, the probiotic assessments, morphological characteristics, sensory evaluation, and survival rates of the covered probiotic cells with Tarkhineh in potato chips during storage time were assessed. Based on results, T34 isolated from traditional Tarkhineh as a safe strain had a high tolerance to low pH and bile salt conditions, displayed acceptable anti-pathogenic activities, and also showed desirable antibiotic susceptibility. Two types of Tarkhineh formulations (plain Tarkhineh and turmeric Tarkhineh) were applied using a simple spraying method for covering T34 cells in potato chips. All formulations showed elliptical to spherical (480-770 μm) shape probiotic drops. Storage stability results revealed that T34 cells mixed with turmeric and plain Tarkhineh during 4 months of storage at 4°C displayed excellent protection abilities with about 3.70 and 2.85 log decreases in CFU/g respectively. Additionally, probiotic potato chips compared to non-probiotic and commercial potato chips, exhibited probiotic product criteria such as excellent quality and superior sensory properties during storage time. In conclusion, Tarkhineh showed high potential as a protective matrix for probiotic cells in potato chips.

## Introduction

Potato chips contain high amounts of starch, fat, dietary fiber, essential micronutrients, and phytonutrients such as potassium, sodium, chlorogenic acid, phenolic acids, and carotenoids (Beals, 2018). On the other hand, due to the change in lifestyle and high demand for potato chips, high consumption in adolescents and young people, researchers are interested in investigating the processing and utilization of this product (Yodkraisri and Bhat, 2012).

During the production process, due to deep frying with oil, the fat content of potato chips is high, which negatively affects the shelf-life and flavor of the final product. On the other hand, due to the process of lipid oxidation during storage, in addition to its harmful effects on human health, the sensory acceptability of the product is reduced. Moreover, owing to the high surface to volume ratio, the oxidative deterioration process in the stored product is high (Abong et al., 2011). To overcome the mentioned problems, a variety of natural and chemical antioxidants have been assessed to add to the processed foods including potato chips (Allam and El-Sayed, 2004). Moreover, modified atmosphere packaging (MAP) with nitrogen has been successfully used to prolong the shelf life of potato chips (Del Nobile, 2001).

This study was performed due to the widespread consumption of potato chips and also the high tendency to use probiotic appetizers (Nami et al., 2019b). Probiotics are scientifically defined non-pathogenic microorganisms that have beneficial health effects when being used consistently and in sufficient doses (Haghshenas et al., 2015c). The majority of probiotics belong to the lactic acid bacteria (LAB) group. *Lactobacillus*, *Bifidobacteria*, and *Streptococcus* are the most famous genera that are described as probiotics (Biradar et al., 2005; Guarner et al., 2008).

A high variety of traditional dairy products, such as Tarkhineh, curd, shiraz, yogurt, and cheese as the main source of beneficial probiotic strains in different parts of Kermanshah province (located in the west of Iran) are produced and consumed (Iran, 2009; Noori et al., 2013). Tarkhineh is a traditional fermented product that is prepared from a mixture of fermented milk, yogurt drink, crushed wheat, spices, and salt (Mehrabian et al., 2012). This product has a high content of vitamins, free amino acids, and minerals (Daglioglu, 2000; Tabatabaei Yazdi et al., 2013). On the other hand, Tarkhineh which can be stored for 1-2 years is a rich source of various probiotic strains, especially LAB groups including *Lactobacillus nagelii* and *Enterococcus facium* (Tabatabaee et al., 2012; Tafvizi and Tajabadi Ebrahimi, 2012).

The challenge in using probiotics is to maintain a significant viable number of bacteria and stability at various heat/moisture conditions in the final product during the storage period. Such challenges can be addressed by covering the cells within a protective matrix material (Nami et al., 2017). Lyophilization is the most common method to preserve probiotics in pharmaceutical and food products. However, the high sensitivity of probiotics to cell water loss during the process and the inefficiency of the method during long storage periods are the chief drawbacks of lyophilization (Mostafa, 2020).

In this research, the potential of different types of Tarkhineh mixtures for their suitability as a coating matrix for probiotic cells during long-term storage was investigated. Two different Tarkhineh formulas (plain Tarkhineh and turmeric Tarkhineh) were mixed with isolated probiotic strains and sprayed through a simple handmade sprayer to produce probiotic drops. For this purpose, we benefited from an easy spraying system that according to the small scale of production is much more feasible, simple, cost-efficient, and higher rates of cell viability when compared to other methods including lyophilization. Besides, using the appropriate concentrations of supporting materials, small probiotic drops can be produced for adding to potato chips. Hence, in this study, the probiotic assessments, morphological characteristics, sensory evaluation, and survival rates of the covered probiotic cells with Tarkhineh in potato chips during storage time were investigated. The aim of this study is initially to isolate, evaluate, and select the most appropriate probiotic strains with health-promoting properties from traditional Tarkhineh. Moreover, we aimed to investigate the effects of Tarkhineh-probiotic drops on the quality of potato chips and the shelf-life of the stored product.

## Materials and Methods

### Sampling, Culture Conditions, and Isolation of Strains

Bacteria strains (Table 1) were isolated from 60 samples of traditional Tarkhineh that were randomly collected from the domestic producers in different diary product suppliers throughout the Kermanshah province of Iran. Tarkhineh samples were transferred to the laboratory separately and stored at 4°C. To isolate bacteria from Tarkhineh samples, 10 g of each sample is suspended in 90 ml of sterile trisodium citrate solution and after one hour, 5 ml of the solution was added to 100 ml of de Man, Rogosa and Sharpe (MRS) broth to enrich and increase the initial population of bacteria (Nami et al., 2019c). The bacteria strains were isolated and amplified through anaerobic growth (anaerobic jar) in MRS broth medium for 24-48 h at 37°C and were spread on MRS agar media similar to the mentioned condition. Then, the colonies were subjected to initial morphological and biochemical tests including cell morphological analyzing test, catalase test, and gram staining (Haghshenas et al., 2015b).

**TABLE 1:** Source, gram staining, catalase test, and survival rates (%) of isolated presumptive LAB after 3 h incubation at pH 2.5 and 4 h incubation at 0.3% bile salt.

Isolates	Source	Gram Staining	Catalase Test	*Survival rates (%) at pH 2.5	*Survival rates (%) at 0.3% bile salt
T1	Tarkhineh	Gram-positive	Catalase-negative	31.02 ± 0.79 ^j^	52.74 ± 2.21 ^f^
T6	Tarkhineh	Gram-positive	Catalase-negative	28.82 ± 1.20 ^k^	39.46 ± 0.62 ^h^
T7	Tarkhineh	Gram-positive	Catalase-negative	88.11 ± 0.73 ^b^	108.64 ± 0.62 ^c^
T12	Tarkhineh	Gram-positive	Catalase-negative	42.51 ± 0.26 ^h^	49.66 ± 1.55 ^g^
T16	Tarkhineh	Gram-positive	Catalase-negative	11.97 ± 0.41 ^o^	22.14 ± 1.76^l^
T19a	Tarkhineh	Gram-positive	Catalase-negative	7.52 ± 1.35 ^p^	16.62 ± 1.81 ^m^
T19b	Tarkhineh	Gram-positive	Catalase-negative	54.68 ± 0.69 ^f^	59.28 ± 1.05 ^de^
T20	Tarkhineh	Gram-positive	Catalase-negative	84.20 ± 0.92 ^c^	205.25 ± 1.94 ^a^
T23	Tarkhineh	Gram-positive	Catalase-negative	43.97 ± 1.74 ^h^	50.76 ± 1.98 ^fg^
T26	Tarkhineh	Gram-positive	Catalase-negative	51.61 ± 0.54 ^g^	58.49 ± 0.88 ^e^
T30	Tarkhineh	Gram-positive	Catalase-negative	60.10 ± 0.73 ^e^	61.80 ± 0.51 ^d^
T34	Tarkhineh	Gram-positive	Catalase-negative	98.35 ± 0.12 ^a^	207.72 ± 1.26 ^a^
T37	Tarkhineh	Gram-positive	Catalase-negative	9.10 ± 0.94 ^p^	20.27 ± 2.74 ^l^
T48	Tarkhineh	Gram-positive	Catalase-negative	78.69 ± 0.82 ^d^	120.82 ± 1.35 ^b^
T55	Tarkhineh	Gram-positive	Catalase-negative	25.79 ± 1.83 ^l^	32.63 ± 1.60 ^i^
T60a	Tarkhineh	Gram-positive	Catalase-negative	17.63 ± 1.15 ^n^	26.03 ± 1.41 ^k^
T60b	Tarkhineh	Gram-positive	Catalase-negative	36.16 ± 0.35 ^i^	40.18 ± 1.10 ^h^
T60c	Tarkhineh	Gram-positive	Catalase-negative	22.82 ± 2.05 ^m^	29.62 ± 1.00 ^j^

*Values followed by the same letters are not significantly different (*P ≤* 0.05). Statistical analysis of each formulation was done separately. Values shown are means ± standard deviations (*n* = 3).

### Low pH and Bile Salt Tolerance Assessments

To evaluate the low pH and high bile salt tolerance, 10 mL of each bacterial culture (1.7–3.9 × 10^9^ CFU/ml) incubated for 24 h in MRS broth, was centrifuged at 4,000 × g for 5 min. The supernatants were removed, and the cell plates were resuspended for 3 h in 10 mL of low pH solution (pH 2.5 at 37°C) and 4 h in 10 ml of high bile salt solution (0.3 % w/v oxgall, pH 6.8, and 37°C) by gentle agitation. The preliminary selection was performed by measuring the optical densities (OD) values at 600 nm according to the method described by Yang et al. (2016). The acid and bile tolerance was estimated by determining the survival rate % as following: [OD (after treatment)/OD (before treatment)] × 100 % (Yang et al., 2016). Afterward, the cells were diluted up to 10 times using sterile saline (sodium chloride: 5.8 g L^–1^) and 100 μL of each dilution was cultured aerobically for 48 h at 37°C on MRS agar medium. The survival rate was calculated using the following equation: survival rate (%) = (log CFU N_1_/log CFU N_0_) × 100 %, in which N_1_ corresponds to the total clones treated with extra acids or bile salts (0.3%) and N_0_ corresponds to the total clones before they were incubated under harsh conditions (Nami et al., 2019a).

### Survival in Simulated Gastrointestinal Digestion

The method described by Seiquer et al. (2010) was performed to evaluate *in-vitro* gastrointestinal digestion. To assess the gastric digestion, pepsin with a final concentration of 5% (w/v) was added to four selected LAB strains (T7, T20, T34, and T48) with initial concentration of 1.7–3.9 × 10^9^ CFU/ml which showed high resistance to low pH and bile salts conditions. Before incubated at 37°C with gentle agitation at 110 rpm for two hours, the pH values were adjusted to 2.5.

To recreate intestinal digestion, solutions of bile salts and pancreatin were added at final concentrations of 0.3 and 0.1% (w/v), respectively. The samples were adjusted to pH 6.0 and incubated at 37°C for three hours with gentle agitation at 110 rpm. Before and after gastric and intestinal digestion, the samples were removed and the aliquots were serially diluted and plated in triplicate on MRS agar. Then, the plates were incubated for 48 hours at 37°C to determine cell count (Seiquer et al., 2010).

### Adhesion to Caco-2 Cells

T7, T20, T34, and T48 LAB strains with high resistance to low pH and bile salts conditions were investigated for their adhesion ability to the human colon carcinoma cell line Caco-2. RPMI medium supplemented with 10% heat-inactivated fetal bovine serum and 1% penicillin-streptomycin mixture were used and cells were cultured on 24-well tissue culture plates and incubated at 37°C in 5% CO_2_ under a relatively humidified atmosphere until a confluent monolayer was formed. Before the adhesion assay, the media in the wells containing a Caco-2 cell monolayer were removed and replaced with fresh antibiotic-free RPMI. Thereafter, 1 × 10^7^ CFU mL^–1^ of bacteria was added to each well with a total volume of 1 mL and then incubated for 3 h at 37°C under an atmosphere of 5% (v/v) CO_2_. The wells were washed twice with a sterile pre-warmed PBS solution to remove non-attached bacterial cells. One mL of 1% (v/v) Triton X-100 was added to each well to detach the cells from the wells and the mixture was stirred for 10 min. To determine the viable cell count, the cell suspension was plated onto MRS agar and incubated at 37°C (Haghshenas et al., 2015c).

### Inhibitory Effects Against Pathogens

The agar diffusion well method was used to determine the antagonistic activity of the isolated strains against some food-borne and clinically important human pathogens such as *Yersinia enterocolitica* (ATCC 23715), *Streptococcus mutans* (PTCC 1683), *Escherichia coli* (PTCC 1276), *Staphylococcus aureus* (ATCC 25923), *Bacillus subtilis* (ATCC 19652), *Listeria monocytogenes* (ATCC 13932), *Klebsiella pneumoniae* (PTCC 1053), and *Shigella flexneri* (PTCC 1234). 1.5 × 10^8^ CFU mL^–1^ (half McFarland) of mentioned pathogens were cultured on Mueller-Hinton agar and then the wells were cut on inoculated medium. The wells were filled with 50 μL of filtered supernatant (overnight cultured) of selected isolates and incubated overnight at 37°C, and finally, the inhibition zone was measured by a digital caliper (Haghshenas et al., 2017).

The nature of anti-pathogenic substances in bacterial extracts was investigated by the method performed by Nami et al. (2019c) with some changes. The cell-free bacterial extracts were obtained by centrifugation at 8,000 × *g* for 20 min at 4°C. Then, after adjusting the pH to 6.2, they were treated for 2 h at 37°C with 1 mg/ml proteinase K (protein nature assessment) and catalase (hydrogen peroxide evaluation), and their anti-pathogenic activities were assessed by a diffusion method.

### Antibiotic Susceptibility Profiles

To determine the antibiotic susceptibility, the disc diffusion method against some highly consumed and clinically important antibiotics such as cefixime (5 μg), azithromycin (15 μg), amoxicillin (25 μg), doxycycline (30 μg), trimethoprim-sulfamethoxazole (1.25/23.75 μg), ciprofloxacin (5 μg), cephalexin (30 μg), amoxicillin-clavulanic acid (20/10 μg), and vancomycin (30 μg) was performed. After overnight incubation of selected isolates on MRS agar medium at 37°C (1.7–3.9 × 10^9^ CFU/ml) and placing antibiotic disks on them, the diameter of the inhibition zone around disks was measured by a digital caliper (Nami et al., 2018b).

### Safety Assessment

#### Hemolytic Activity

The hemolytic activity of isolates was assessed according to Nami et al. (2019c). Three categories were used for the detection of hemolytic activity: a clear halo around the colony for β-hemolysis; a greenish halo for α-hemolysis, and no halo for γ-hemolysis.

### Detection of Virulence Genes

Multiplex PCR was carried out to detect potential virulence genes. Thirteen virulence genes were investigated in this study (Table 2) and *Enterococcus faecium* ATCC 8043 and *Enterococcus faecalis* ATCC 29212 were used as control. Multiplex PCR was performed based on this program: 95°C for 5 min as initial denaturation followed by 35 cycles of denaturation (95°C for 60 s), annealing at 54°C and 56°C for 60 s, elongation at 72°C for 60 s, and final elongation at 72°C for 5 min (Table 2). The PCR products were visualized using 3% agarose gel.

**TABLE 2:** Primers used for PCR amplification of virulence factors.

Gene	Responsible for	Sequence (5′-3′)	Ta (°C)	Amplicon size (bp)	References	PCR
*esp*	Immune invasion	F: TTACCAAGATGGTTCTGTAGGCAC R: CCAAGTATACTTAGCATCTTTTGG	56	510	Nami et al., 2015	**Multiplex PCR Program 1**
*ace*	Adhesion of collagen	F: AAAGTAGAATTAGATCCACAC R: TCTATCACATTCGGTTGCG	56	320	Nami et al., 2015	
*ccf*	Sex pheromone	F: GGGAATTGAGTAGTGAAGAAG R: AGCCGCTAAAATCGGTAAAAT	56	543	Nami et al., 2015	
*gel E*	Hydrolysis of gelatin, collagen, and hemoglobin	F: ACCCCGTATCATTGGTTT R: ACGCATTGCTTTTCCATC	56	419	Nami et al., 2015	
*cylA*	Cytolysin	F: TGGATGATAGTGATAGGAAGT R: TCTACAGTAAATCTTTCGTCA	56	517	Nami et al., 2015	
*cylM*	Cytolysin	F: CTGATGGAAAGAAGATAGTAT R: TGAGTTGGTCTGATTACATTT	54	742	Eaton and Gasson, 2001	**Multiplex PCR Program 1**
*cylB*	Cytolysin	F: ATTCCTACCTATGTTCTGTTA R: AATAAACTCTTCTTTTCCAAC	54	843	Eaton and Gasson, 2001	
*agg*	Cell aggregation	F: AAGAAAAAGAAGTAGACCAAC R: AAACGGCAAGACAAGTAAATA	54	1553	Eaton and Gasson, 2001	
*cpd*	Sex pheromone	F: TGGTGGGTTATTTTTCAATTC R: TACGGCTCTGGCTTACTA	54	782	Eaton and Gasson, 2001	
*cob*	Sex pheromone	F: AACATTCAGCAAACAAAGC R: TTGTCATAAAGAGTGGTCAT	54	1405	Eaton and Gasson, 2001	

*Ta (°C): Annealing temperature; bp: base pairs.*

### Molecular Identification

The total genomic DNA was extracted according to Nami et al. (2018a). F: 5′-AGAGTTTGATCMTGGCTCAG-3′ and R: 5′-TACCTTGTTAGGACTTCACC-3′ primers were used to amplify the 16S-rRNA gene. The PCR program cycles performed as follows: denaturation at 94°C for 4 min, 32 cycles of 94°C, 60 s, 58°C, 60 s, 72°C for 60 s, and the final extension was performed for 5 min in 72°C. The purified DNAs were sequenced by the Korean sequencing company, Macrogene. The sequencing results were blasted with the deposited sequences in the NCBI and GenBank site^1^ to identify the isolated bacterial strains.

### Potato Chips Preparation

Potato (*Solanum tuberosum* var. Ramoae) was purchased from the local market, Kermanshah, Iran. Soybean oil was obtained from Nazgol Company (Mahidasht, Kermanshah, Iran). The potato tubers were washed well and after peeling, they were cut into thin slices (1-2 mm). Then, for more crispness, they were soaked in CaCl_2_ solution (1%) for 15 min. After the refined soybean oil reached a temperature of 200 °C in a stainless steel pan, the potato slices were fried for 5-7 minutes and cooled after taking the extra oil (Mostafa, 2020).

### Preparation of Probiotic Cell Culture

The isolated probiotic cells were grown by anaerobic growth of 100 μL respective stock cultures in 15 mL MRS medium for 24 h at 37°C. The cells were harvested by centrifugation (10 min, 1200 × *g*) at 4°C, washed, and resuspended in phosphate buffer (pH 7.2). The cells were counted to a desirable amount (>10^10^ CFU/g) three times in MRS agar using the pour plate method, before the preparation step. The equal volume of the viable cell population was divided and used in the preparation step by different Tarkhineh blends (Nami et al., 2018a).

### Probiotic Tarkhineh Preparation and Chemical Analysis

First, wheat semolina (0.5 kg) was washed and soaked in diluted buttermilk or yogurt (1.5 L) for 3 to 4 h, and then salt (10 g), dried mint, and pennyroyal (5 g) were added. The mixture was poured into two separate pots. The pots were then placed on the heat and stirred constantly until they boiled and become thick. Inside one of the pots (turmeric Tarkhineh), 10-gram turmeric was added and the other pot was prepared as plain Tarkhineh. The solutions were stirred well until the buttermilk was absorbed into the wheat semolina and became relatively pasty. After the Tarkhineh was cooled, it was completely homogenized and finely crushed by a mixer. Certain amounts of both plain and turmeric Tarkhineh were separately added in 50 ml falcons and after autoclaving and under the safety cabinet were inoculated with selected probiotic bacteria and then were kept in an incubator at 37°C until being used (Vasiee et al., 2014). The difference between the weight of Tarkhineh pastes before and after drying on the weight of Tarkhineh paste before drying was considered as the percentage of moisture. The pH level of the Tarkhineh paste was measured using a pH meter. The percentage of calcium and phosphorus was estimated by spectrophotometry. The amounts of protein, salt, ash, and fat were measured using the method described by Mashak et al. (2014). Finally, the percentage of carbohydrates was obtained by reducing the total weight of solids from the weight of fat, salt, protein, and ash (Mashak et al., 2014).

### Probiotic Potato Chips Production, Morphological Analysis, and Spraying Efficiency

The potato slices were placed in a completely sterile environment in special polyethylene bags and then the probiotic Tarkhineh containing a certain number of lactic acid bacteria (10^10^ CFU/g) was sprayed on the surface of the potato slices in small drops by a handmade sprayer. The Tarkhineh paste containing probiotics was transferred through a sterile tank by a pump to the sprayer head at the end of the machine to turn into fine liquid droplets. Under the laminar flow cabinet, the droplets were sprayed on the surface of the potato chips and then dried (Mostafa, 2020).

The size of probiotic drops was measured using a laser diffraction particle size analyzer (Mastersizer 3,000, Malvern Instruments, United Kingdom). The average size of the drops was estimated from the mean diameter of 50 drops obtained from each potato chips samples. The morphology of probiotic drops was investigated using scanning electron microscopy (Hitachi SU3800). For better conductivity during imaging, samples were covered for 500 s with an ultrathin layer of gold (thickness of approximately 5–6 nm) by a sputter coater.

To determine the spraying efficiency, 50 mg of probiotic drops was poured in 5 mL phosphate buffer (pH 7.2) at 37°C for 20 min and subsequently, the entrapped viable probiotic cells were counted by the pour plate technique in MRS agar. The spraying efficiency was calculated by the following equation:

Spraying efficiency (SE) = (Log_10_N/Log_10_N_0_) × 100.

Where N is the number of entrapped viable probiotic cells and N0 displays the free viable probiotic cells before spraying (Lotfipour et al., 2012).

### Storage Stability

The stability of probiotic bacteria was assessed during 120 days of storage in vacuum-packed polyethylene bags at room (25°C) and refrigerated (4°C) temperature in a dark place. The viability of cells in seven different storage times (0, 20, 40, 60, 80, 100, and 120 d) were measured. During storage time, 1 g probiotic potato chips at room temperature was dissolved in 10 ml sodium citrate solution (50 mM) with pH 7.5 by gentle shaking (100 rpm). The released probiotic cells were serially diluted 10 times using saline solution, and then, 50 μl of aliquots were placed on the MRS agar for 24 h anaerobic growth (37°C). The viable (%) rates of probiotic cells were calculated by utilizing the pour plate method in MRS agar. Meanwhile, potato chips containing lyophilized probiotic cells with 10% skim milk + 5% sucrose (SM) were used as controls (Haghshenas et al., 2015d; Mostafa, 2020).

### Sensory Evaluation

A trained panel of 15 students and staff of the faculty of pharmacy, Kermanshah University of medical sciences (Kermanshah, Iran) evaluated probiotic, non-probiotic, and commercial potato chips samples in terms of various sensory characteristics such as color, smell, appearance, texture, taste, and general acceptance. Samples were evaluated for sensory evaluation using a 9-point hedonic scale (from 9 to 1) while, 9 was very pleasant and 1 was very unpleasant (Joshi et al., 2016).

### Statistical Analysis

All experiments were designed based on a completely randomized design with three replications for each experimental group. Then the data were analyzed using ANOVA and Duncan statistical tests and *p* ≤ 0.05 was considered to be statistically significant. SPSS statistics19 software was used in data analysis (Casler, 2015).

## Results

### Bacterial Morphological and Biochemical Analysis

The whitish to creamy hemispherical shape bacterial colonies were isolated. From the existing colonies, a total of 18 rod or spherical shape bacteria that were catalase-negative and gram-positive and grown in a specific culture medium (MRS) under anaerobic conditions were isolated as presumptive LAB bacteria. These 18 isolates were selected and examined for further analysis.

### Low pH and Bile Salt Tolerance Assessments

Optical density (OD) analysis at pH 2.5 for 3 h and 0.3% bile salt for 4 h was used as a preliminary assessment for screening 18 LAB strains. The results are shown in Table 1. The results revealed that some of the tested strains survived well in acidic and bile environments. On the other hand, there was a large difference in the survival of the strains under artificially harsh conditions. According to the preliminary OD results, four strains including T7, T20, T34, and T48 had survival rates of more than 78% and were therefore selected for further analysis.

Log CFU mL^–1^ for selected LAB strains after 3 h of incubation at pH 2.5 and 4 h of incubation at 0.3% oxgall is shown in Table 3. After 1 h of incubation under harsh conditions, slight decreases in log CFU (≤0.454) were observed and all strains showed high survival rates in the first hour of incubation. From hour 1 to hour 2, greater decreases in log CFU (0.191-3.009) were observed, and T34 and T7 strains showed a higher level of resistance to the acidic and bile salt conditions than the T20 and T48 strains. Between hour 2 to hour 3 all four strains showed a slight decrease in log CFU (≤0.404). Finally, from hour 3 to hour 4 under high bile salt condition, a very slight decrease in log CFU (0.039-0.128) was observed among the strains.

**TABLE 3:** Re-screening results and survival rates (%) of isolated LAB after 3 h incubation at pH 2.5 and 4 h incubation at 0.3% bile salt.

Isolates	Final counts (log CFU/ml) after 3h incubation at pH 2.5					Final counts (log CFU/ml) after 4h incubation at 0.3% bile salt					
	0 h	1 h	2 h	3 h	*SR (%)	0 h	1 h	2 h	3 h	4h	*SR (%)
T7	9.43 ± 0.21	9.19 ± 0.16	8.01 ± 0.19	7.82 ± 0.23	83.12 ± 0.22^b^	9.23 ± 0.27	9.14 ± 0.22	8.72 ± 0.15	8.64 ± 0.21	8.59 ± 0.20	93.17 ± 0.24^b^
T20	9.59 ± 0.27	9.14 ± 0.24	7.32 ± 0.19	7.19 ± 0.25	75.17 ± 0.26^c^	9.59 ± 0.37	9.24 ± 0.31	8.76 ± 0.31	8.62 ± 0.39	8.58 ± 0.27	89.29 ± 0.31^c^
T34	9.23 ± 0.31	8.79 ± 0.25	8.12 ± 0.29	7.94 ± 0.14	86.25 ± 0.31^a^	9.53 ± 0.34	9.52 ± 0.33	9.33 ± 0.30	9.29 ± 0.28	9.25 ± 0.26	97.16 ± 0.25^a^
T48	9.53 ± 0.28	9.23 ± 0.25	6.22 ± 0.18	5.81 ± 0.17	61.27 ± 0.26^e^	9.43 ± 0.29	9.21 ± 0.32	8.74 ± 0.26	8.52 ± 0.27	8.39 ± 0.29	89.09 ± 0.18^c^

*Values followed by the same letters are not significantly different (*P ≤* 0.05). Statistical analysis of each formulation was done separately. SR: Survival Rate; Values shown are means ± standard deviations (*n* = 3).

### Survival in Simulated Gastrointestinal Digestion

A total of four selected LAB (most resistant strains to low pH and bile salts conditions) were tested further through a simulated digestion test. All four strains including T7, T20, T34, and T48 survived after exposure to the simulated digestion conditions. The highest percentage of survivability was observed for T34 with a survivability value of 68%, followed by T48, T20, and T7, with survival values of 59, 41, and 32 respectively.

### Adhesion to Caco-2 Cells

All four selected strains were examined for their ability to adhere to Caco-2 cells. Based on results, strain T34 was the most adherent strain, with an adhesion value of 4.5 × 10^6^ CFU mL^–1^, followed by strains T48 and T20, with adhesion values of 2.3 × 10^6^ and 3.8 × 10^5^ CFU mL^–1^, respectively. Strain T7 was not able to adhere to Caco-2 cells.

### Inhibitory Effects Against Pathogens

Isolates T7, T20, T34, and T48 had an acceptable tolerance to low pH and high bile salt conditions. Therefore, they were selected to evaluate their inhibitory effects on pathogens. The antagonistic activities of 4 isolated LAB against 8 pathogens are shown in Table 4. The results showed that three isolates including T20, T34, and T48 displayed significant anti-pathogenic activities against indicator microorganisms and were able to inhibit the growth of all pathogens. By contrast, T7 exhibited moderate antagonistic activity and inhibited the growth of three pathogens including *Y. enterocolitica*, *S. mutans*, and *S. aureus* (Table 4). Similar to our results, the high antagonistic activities for LAB isolates against the high diversity of pathogenic bacteria were reported (Martinez et al., 2013; Settanni et al., 2014).

**TABLE 4:** The inhibitory effect of isolated LAB strains against pathogens. Values shown are means ± standard deviations (*n* = 3).

	Indicator pathogens Diameter of inhibition zone (mm)							
Isolates	*Y. enterocolitica*	*S. mutans*	*E. coli*	*S. aureus*	*B. subtilis*	*L. monocytogenes*	*K. pneumoniae*	*S. flexneri*
T7	9.6 ± 0.7^c^*	5.4 ± 0.1^b^	0.0 ± 0.0^d^	5.8 ± 0.6^b^	0.0 ± 0.0^c^	0.0 ± 0.0^b^	0.0 ± 0.0^c^	0.0 ± 0.0^c^
T20	13.1 ± 0.6^a^	10.4 ± 0.7^a^	16.1 ± 0.6^a^	10.7 ± 0.7^a^	12.6 ± 0.7^a^	12.2 ± 0.4^a^	9.4 ± 0.8^b^	11.5 ± 0.9^a^
T34	12.3 ± 0.8^ab^	10.8 ± 0.2^a^	12.5 ± 0.8^b^	10.5 ± 0.7^a^	12.4 ± 1.1^a^	12.5 ± 0.2^a^	9.4 ± 1.0^b^	12.2 ± 0.6^a^
T48	11.6 ± 0.8^b^	11.1 ± 0.3^a^	10.8 ± 0.3^c^	11.2 ± 0.3^a^	9.6 ± 0.8^b^	12.9 ± 1.0^a^	12.1 ± 0.4^a^	10.2 ± 0.6^b^

*Values are mean ± standard error of triplicates. ^*a–d*^ Means in the same row with different lowercase letters differed significantly (*P ≤ 0.05*). S (strong *r* ≥ 20 mm), M (moderate *r* < 20 mm and > 10 mm), and W (weak ≤ 10 mm).

After adjusting the pH of the bacterial extracts to 6.2, isolates T7 and T48 could not show any anti-pathogenic properties. Also, isolates T20 and T34 were not able to inhibit the growth of *L. monocytogenes*, *S. flexneri*, *B. subtilis*, and *K. pneumoniae*. Therefore, it is concluded that the nature of the inhibition of these isolates against the mentioned pathogens is due to acid production. On the other hand, after treating the extracts of T20 and T34 isolates with catalase enzyme and performing anti-microbial tests against *Y. enterocolitica*, *S. mutans*, *E. coli*, and *S aureus*, no bacterial extract was able to inhibit the growth of indicator pathogens, except for isolate T20 against *Y. enterocolitica* and *E. coli* and isolate T34 against *S. aureus*. Therefore, it was concluded that the inhibitory nature of those isolates against the mentioned pathogens is due to the production of hydrogen peroxide. Finally, the bacterial extracts were treated with protease K enzyme, and the antimicrobial properties of T20 and T34 isolates against *Y. enterocolitica*, *E. coli*, and *S. aureus* were investigated. The results showed that no inhibition zone was observed, indicating the bacteriocin nature of their extracts against the mentioned pathogens.

### Antibiotic Susceptibility Profiles

Based on the previous results, T20, T34, and T48 had a high tolerance to low pH and bile salt conditions and also displayed acceptable anti-pathogenic activities. Therefore, based on these results and hemolytic activity, these 3 isolates were selected for antibiotic susceptibility evaluation. The antibiotic susceptibility results of isolated LAB against nine clinically important and widely used antibiotics in Iran are presented in Table 5 (Abbasian et al., 2019). All three isolates were sensitive or semi-sensitive to cefixime, azithromycin, amoxicillin, doxycycline, cephalexin, and amoxicillin-clavulanic acid.

**TABLE 5:** Antibiotic susceptibility profiles of isolated LAB.

	Antibiotics susceptibility zone of inhibition (mm)								
Isolated Strains	CFM	AZM	AMX	D	SXT	CP	CN	AMC	V
T20	22 S	15 I	29 S	26 S	26 I	0 R	24 S	31 S	0 R
T34	18 I	14 I	28 S	28 S	28 I	0 R	21 S	29 S	0 R
T48	28 S	20 S	30 S	26 S	20 R	0 R	25 S	32 S	0 R

*CFM, cefixime; AZM, azithromycin; AMX, amoxicillin; D, doxycycline; SXT, trimethoprim-sulfamethoxazole; CP, ciprofloxacin; CN, cephalexin; AMC, amoxicillin-clavulanic acid; V, vancomycin. Cefixime results based on R ≤ 15 mm; I: 16–18 mm; S ≥ 19 mm. Azithromycin results based on R ≤ 13 mm; I: 14–17 mm; S ≥ 18 mm. Amoxicillin results based on R ≤ 18 mm; I: 19–21 mm; S ≥ 22 mm. Doxycycline results based on R ≤ 10 mm; I: 11–13 mm; S ≥ 14 mm. Trimethoprim-sulfamethoxazole results based on R ≤ 25 mm; I: 26–29 mm; S ≥ 30 mm. Ciprofloxacin results based on R ≤ 15 mm; I: 16–20 mm; S ≥ 21 mm. Cephalexin results based on R ≤ 14 mm; I: 15–17 mm; S ≥ 18 mm. Amoxicillin-clavulanic acid results based on R ≤ 13 mm; I: 14–17 mm; S ≥ 18 mm. Vancomycin results based on R ≤ 14 mm; I: 15–16 mm; S ≥ 17 mm. (Performance Standards for Antimicrobial Susceptibility Testing, from Clinical and Laboratory Standards Institute, Twenty-Third Informational Supplement, Wayne, PA (CLSI 2013)).*

### Safety Assessment

#### Hemolytic Activity

Based on results, isolate T7 showed α-hemolytic activity, while isolates T20, T34, and T48 showed no hemolytic activity. None of the tested isolates showed β-hemolytic activity.

#### Detection of Virulence Factors

The presence of genes encoding ten known virulence factors in the tested isolates was assessed. The results of PCR amplification revealed that strain T34 harbored none of the tested virulence factors; while strain T20 showed the presence of *esp* gene and strain T48 harbored *ace*, *ccf* and, *cpd* genes.

### Molecular Identification and Probiotic Characterization

The PCR-amplified fragments of the 16S-rRNA genes of the isolates were sequenced. Based on the results, isolate T20 belonged to *Enterococcus durans* (Accession No. MW433680), isolate T34 belonged to *Lactococcus lactis* subsp. *cremoris* (Accession No. MW433677) and isolate T48 belonged to *Enterococcus faecalis* (Accession No. MW433678).

### Probiotic Potato Chips Production, Chemical, and Morphological Analysis, and Spraying Efficiency

Two types of probiotic potato chip formulations (turmeric and plain Tarkhineh) were designed and produced to improve storage stability and enhance the sensory properties of the T34 probiotic strain. T34 strain as a safe and adherent strain had a high tolerance to low pH and bile salt conditions, displayed acceptable anti-pathogenic activities, and also showed desirable antibiotic susceptibility. According to experiments, low concentrations (less than 25% (w/v)) of Tarkhineh paste used as a probiotic supporting companion resulted in reduced viscosity and did not create uniform probiotic drops. On the other hand, spraying of Tarkhineh paste through a sprayer head at high concentrations (more than 35% (w/v)) was difficult due to high viscosity. Hence, the present study selected 30% (w/v) Tarkhineh powder as the optimal concentration for the probiotic support matrix.

Turmeric and plain Tarkhineh pastes had high moisture contents (73.40 and 73.81 g 100g^–1^ respectively). Measuring the pH of the Tarkhineh samples showed that they had a pH spectrum of 4.83–4.94. The amount of calcium and phosphorus were 326 and 268 mg 100g^–1^ for turmeric Tarkhineh and 321 and 267 mg 100g^–1^ for plain Tarkhineh. The crude protein, salt, ash, and fat contents for turmeric Tarkhineh were 4.14, 2.02, 2.45, and 1.04 g 100g^–1^ and for plain Tarkhineh were 4.11, 2.01, 2.44, and 1.02 g/100g respectively. Finally, the carbohydrate content of turmeric and plain Tarkhineh paste were 16.35 and 16.02 g 100g^–1^ respectively.

**FIGURE 1:**
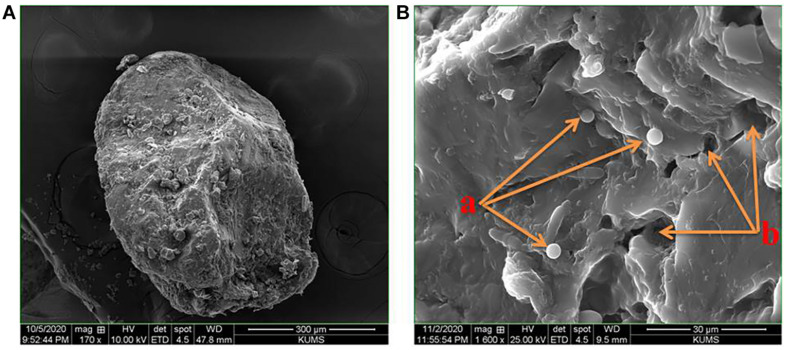
Morphological images of T34 probiotic drop with Tarkhineh texture by the scanning electron microscopy (Hitachi SU3800): **(A)** elliptical shape probiotic drop and their protective layers for the probiotic cells, **(B)**: (a) network-like structures of Tarkhineh texture and entrapped probiotic cells and (b) tiny pores on the surface of Tarkhineh texture.

The morphological analysis by scanning electron microscopy revealed that probiotic drops with various Tarkhineh formulations had almost elliptical to spherical shapes and Tarkhineh texture acts as a protective layer for the probiotic cells (Figure 1A). Moreover, the microscopy images demonstrated that in probiotic drops, Tarkhineh particles were integrated into network-like structures and entrapped the probiotic cells (Figure 1B (a)). Moreover, the SEM images showed that on the surface of all the Tarkhineh formulations, there are a lot of tiny pores (Figure 1B (b)).

The average diameters (based on 50 drops) for probiotic drops were 480–770 μm. The mean diameters of drops containing turmeric Tarkhineh were not significantly (*P ≤* 0.05) different than drops containing plain Tarkhineh.

High spraying efficiency with a range of 98.6–99.4% was observed for trapped probiotic cells. The results showed that bacterial cells were successfully (> 98%) entrapped in the prepared drops. According to the results, no significant differences were observed for spraying efficiency of probiotic cells among these two Tarkhineh formulations, therefore, the spraying efficiency was formulation-independent.

#### Storage Stability

**FIGURE 2:**
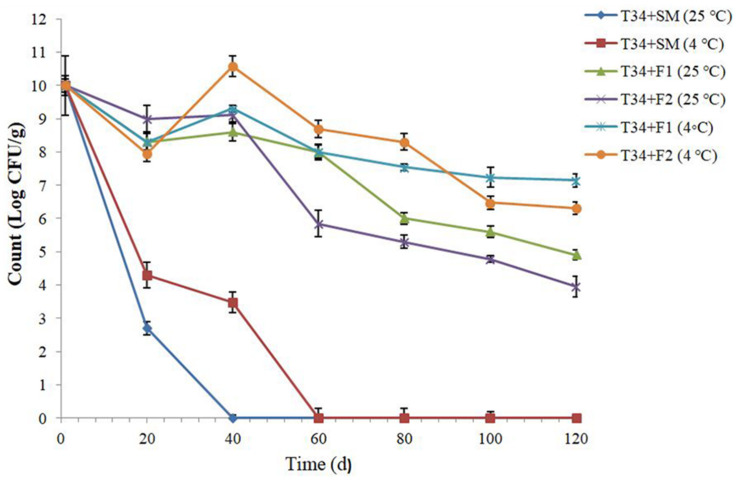
Storage stability of lyophilized and covered T34 probiotic strain with different Tarkhineh formulations during 120 days storage at 25°C and 4°C. T34: *Lactococcus lactis*. F1: plain Tarkhineh. F2: turmeric Tarkhineh. SM: 10% skim milk + 5% sucrose. Values shown are means ± standard deviations (*n* = 3).

The covered T34 in two Tarkhineh formulations (F1: plain Tarkhineh and F2: turmeric Tarkhineh) displayed significantly high cell viabilities (*P* ≤ 0.05) at the storage time at 25°C and 4°C. The T34 cells blend with turmeric Tarkhineh (T34 + F2) and plain Tarkhineh (T34 + F1) at 25°C had week protective ability with around 6.05 and 5.10 log decrease in CFU g^–1^ respectively. Besides, T34 cells blend with turmeric Tarkhineh at 4°C (T34 + F2) showed moderate protective ability with a 3.70 log decrease in CFU g^–1^. But the excellent viability of covered cells was observed for T34 cells blend with plain Tarkhineh at 4°C (T34 + F1) with a 2.85 log decrease in CFU g^–1^ (Figure 2).

The probiotic formulations at the low temperature (4°C) had significant (*P* ≤ 0.05) higher protective abilities compared to the higher temperature (25°C). Moreover, the addition of turmeric to Tarkhineh formulations, especially at 25°C, significantly (*P* ≤ 0.05) reduced the viability of covered cells (Figure 2).

#### Sensory Evaluation

Based on the results of storage stability, T34 probiotic formulations at refrigerated temperatures showed acceptable protective abilities. Therefore, a sensory evaluation was performed after 4 months of storage at 4°C. The mean values of the sensory characteristics such as color, smell, appearance, texture, taste, and general acceptance for various probiotic potato chips formulations are shown in Table 6. No other commercial or non-probiotic potato chips were chosen as control. At the end of storage time, sensory evaluation scores showed a significant difference (*P* ≤ 0.05) between probiotic and control potato chips. Based on the results, probiotic potato chips with different formulations (plain Tarkhineh and turmeric Tarkhineh) obtained acceptable scores in terms of sensory evaluations compared to control samples (Table 6). Probiotic potato chips covered with plain Tarkhineh and turmeric Tarkhineh (T34 + F1 and T34 + F2) had the highest smell, color, and general acceptance scores, but these sensory scores were the lowest in control samples (non-probiotic potato chips and commercial potato chips). On the other hand, in two sensory parameters (taste and texture) there was no significant difference (*P* ≤ 0.05) between probiotic and non-probiotic samples (Table 6).

**TABLE 6:** The sensory evaluation (smell, color, taste, texture, appearance, and general acceptance) of non-probiotic potato chips, commercial potato chips, and probiotic potato chips with different prepared formulations (Tarkhineh) during 120 days storage at 4°C. T34: *Lactococcus lactis*. F1: plain Tarkhineh. F2: turmeric Tarkhineh. Values shown are means ± standard deviations (*n* = 3).

	Score Designated (1 to 9)					
Formulation	Smell	Colour	Taste	Texture	Appearance	General Acceptance
T34 + F1	6.87 ± 1.00^b^*	5.67 ± 0.72^b^	6.27 ± 1.71^a^	7.00 ± 0.84^a^	6.53 ± 0.74^b^	7.87 ± 0.83^a^
T34 + F2	8.27 ± 0.59^a^	8.40 ± 0.74^a^	7.47 ± 1.06^a^	7.47 ± 1.06^a^	7.33 ± 0.90^a^	8.20 ± 0.77^a^
Non-probiotic chips	4.80 ± 0.86^c^	6.00 ± 0.53^b^	6.33 ± 1.76^a^	7.27 ± 0.88^a^	6.47 ± 0.74^b^	5.33 ± 0.82^c^
Commercial chips	4.87 ± 0.83^c^	5.80 ± 0.77^b^	7.00 ± 2.00^a^	7.67 ± 0.90^a^	7.87 ± 0.64^a^	6.67 ± 0.72^b^

*Values are mean ± standard error of triplicates. ^*a–c*^ Means in the same row with different lowercase letters differed significantly (*P* ≤ 0.05).

## Discussion

Oral probiotics must pass through the body’s defense systems, including low pH and bile salts, to colonize the gastrointestinal tract and show health-promoting effects (Sahadeva et al., 2011). *In vitro* and *in vivo* resistance tests show the same cell viability results. Therefore, the tolerance of bacteria to gastrointestinal conditions can be evaluated by *in vitro* methods with the same pH (2.5 for 3 h) and oxgall concentration [0.3% (w / w) for 4 h] (Haghshenas et al., 2015c).

The moderate survival rates in harsh conditions from 61 to 89% were observed in T20 and T48 strains. Also, the T7 strain showed higher survival rates which were 83% at low pH and 93% at bile salt conditions. Also, the T34 strain had the best results and showed high tolerance to harsh conditions (86 -97%). Other studies similar to our results have shown that tolerance to harsh conditions in LAB bacteria is high and strain-specific due to their bilayer membrane structure (Park et al., 2006; Musikasang et al., 2009). All 4 isolates displayed high tolerance to bile salt conditions, ranging from 10 to 28% higher than their low pH tolerance. Bile salts show less lethal effects on bacterial cells than low pH due to their stress adaptation mechanisms in acidic conditions (Begley et al., 2005; Sahadeva et al., 2011). Based on the results, all selected four LAB strains (T7, T20, T34, and T48) retained their viability even after exposure to low pH and high bile salt environments. As a result, these four isolates were selected for further analysis.

One of the most important criteria for bacteria to be distinguished as a probiotic is their ability to remain alive while passing through the upper digestive tract to reach the large intestine, where their useful actions are expected. Similar to the results of selected four LAB strains, high survival rates were reported for probiotic strains including *L. plantarum* 15HN, *L. lactis* subsp. *cremoris* 44L, *E. faecalis* 13C, *E. mundtii* 50H, and *E. durans* 39C in simulated digestion condition by Haghshenas et al. (2015d).

To be colonized in the intestine, probiotic bacteria have to adhere to the intestinal mucosa to avoid being removed from the colon by peristalsis. Our results are in accordance with that of other studies, which showed that LAB strains could adhere well to Caco-2 cells (Ramos et al., 2013).

Possessing an acceptable inhibitory function against pathogens is an expected property of probiotic bacteria, so for this purpose, the antagonistic activities of four selected isolates against 8 gram-positive and gram-negative pathogens were assessed (Cizeikiene et al., 2013). Probiotics belonging to the LAB group mainly inhibit the growth and spread of bacterial pathogens through a combination of various antimicrobial mechanisms such as the production and secretion of hydrogen peroxide (H_2_O_2_), organic acids (lactic acid), and inhibitory proteins (bacteriocin) (Cizeikiene et al., 2013). Our findings showed that the anti-pathogenic activities of T7, T20, T34, and T48 strains are mainly linked to acidification capability and secretion of hydrogen peroxide (Nami et al., 2019c). On the other hand, antagonistic activity due to bacteriocin secretion was observed against a limited number of gram-negative and positive pathogens (Izquierdo et al., 2008). These results are in contrast with other studies reporting that LAB bacteriocins are effective only on gram-positive pathogens and have no effect on gram-negative pathogens due to their outer membranes (Stevens et al., 1991; Zommiti et al., 2018).

Some pathogens, such as *Y. enterocolitica* and *K. pneumoniae*, in addition to high prevalence, show high resistance to antibiotics. Therefore, LAB strains isolated from safe dairy sources similar to our results can be used to neutralize antibiotic-resistant gram-negative pathogens (Shoma et al., 2014; Fàbrega et al., 2015).

Overuse of antibiotics has led to the widespread emergence of antibiotic resistance genes in probiotic bacteria, which can transmit resistance genes to other microorganisms in the gastrointestinal tract and cause acute problems. Therefore, the susceptibility to antibiotics is a fundamental characteristic of probiotic selection (Temmerman et al., 2003). High sensitivity to antibiotics in isolated strains (T20, T34, and T48) is probably due to the limited use of animal antibiotics in the rural area of Kermanshah province. In contrast to our results, the high resistance to mentioned antibiotics among the LAB bacteria was reported by other researchers (Coppola et al., 2005; Neut et al., 2017; Selvin et al., 2020). All three isolated strains resisted ciprofloxacin and vancomycin. On the other hand, resistance to trimethoprim-sulfamethoxazole was observed only in T48. Different strains of the LAB group such as the *Lactobacillus* genus carry the trimethoprim-sulfamethoxazole, ciprofloxacin, and vancomycin resistance genes which confirm our results (Bernardeau et al., 2008; Noor Uddin et al., 2015).

One of the bacterial toxins expressed by some *Enterococcus* strains is *cytolysin*. Therefore, the absence of cytolysin-encoding genes is a desirable feature for enterococci used in the food industry. In this research, only isolate T7 had cytolysin-encoding genes (*cylA* and *cylB*). Besides, only isolate T7 showed the presence of *agg* and *ccf* genes that encoding virulence factors. The results are in accordance with the results of Liu et al. (2015) and Nami et al. (2019c).

Overall, the presence of virulence genes is higher in *E. faecalis* strains than in other enterococci, which is consistent with our results.

Similar probiotic drops with protective networks were observed by Haghshenas et al. (2015d) and Lotfipour et al. (2012). Spherical or elliptical shapes of probiotic drops facilitate their industrial production and give a better appearance to probiotic products (Chan, 2011; Wang et al., 2013).

The mean diameters of drops containing turmeric Tarkhineh were not significantly (*P ≤* 0.05) different than drops containing plain Tarkhineh suggesting that the addition or absence of turmeric has no effect on the size of probiotic drops. The small sizes of drops in probiotic formulations same as our results, do not change the structure and texture of products and can easily be consumed and prescribed (Truelstup-Hansen et al., 2002). On the other hand, according to Sohail et al. (2011), the smaller sizes of probiotic drops (10–40 μm) were observed (Sohail et al., 2011). These high variations in the sizes of drops can be due to different compositions and concentrations of probiotic companions (Voo et al., 2011).

The minimum standard for viable probiotics in food products is 10^6^ CFU g-1. Therefore, trapped probiotic cells must have high spray efficiency to exhibit health-promoting effects. Because of the high spraying efficiency (>98%) observed in our results, the efficient viable cells (≥10^8^ CFU mL^–1^) can be released (Krasaekoopt et al., 2006).

Probiotic bacteria have several problems to survive during storage time in food products. Therefore, to overcome this, suitable storage conditions and compatible protective environments must be selected. In recent studies, covering environments such as whey, skim milk, glucose, sucrose, and glycerol have been used to protect probiotic cells (Guergoletto et al., 2012). The probiotic carriers such as potato chips are usually stored at room temperature (25°C) or refrigerator (4°C) for at least 4 months. Hence, stability tests were performed under the mentioned conditions (Colakoglu and Gursoy, 2011; Mostafa, 2020). The probiotic cells in potato chips were counted every 20 days for up to 120 days at 25°C and 4°C.

Log CFU g^–1^ for lyophilized and covered T34 probiotic cells with different Tarkhineh formulations during the storage time in potato chips are shown in Figure 2. Lyophilized T34 cells displayed a dramatic decrease in their cell viability during four-month storage at 25°C and 4°C. Their cell viability dropped from 10.00 to 0.00 log CFU g^–1^ after 40 days and 60 days storage at 25°C and 4°C respectively. The greatest rates of decrease were found in the first twenty days, while in the next days the decreases with a low slope were observed. This decreasing trend was probably due to the temperature shock in the first twenty days and the subsequent adaptation process in the remaining days of storage. The same results were observed by Mostafa (2020) where the cell viability of lyophilized *Bifidobacterium longum* and *Lactobacillus helveticus* in potato chips after two months of storage dropped from 10.14 to 0.00 log CFU g^–1^ and 9.90 to 0.00 log CFUg^–1^ respectively (Mostafa, 2020).

Same as our results the high cell viability rates after storage at the low temperatures (4°C) were observed for covered probiotics in prebiotic-based hydrogels (Chávarri et al., 2010; Haghshenas et al., 2015a; Nami et al., 2017). However, the viability of probiotic cells blend with turmeric Tarkhineh is significantly reduced compared to simple Tarkhineh. Various studies, such as our findings, have proven the antimicrobial effects of turmeric (Zorofchian Moghadamtousi et al., 2014; Gul and Bakht, 2015).

The excellent protective ability for Tarkhineh-based formulations can be explained by the dense network structure of probiotic drops and the high growth stimulation activities (prebiotic) of its ingredients (wheat semolina and buttermilk). In this study, the use of higher concentrations of Tarkhineh (>30% (w/v)) could create denser network structures and lead to better protection and growth, but extrusion of Tarkhineh paste with high concentrations through sprayer head was difficult and reduced the production efficiency.

Distinctive characteristics of potato chips including taste, appearance, and flavor have a great impact on their production and marketability (Halagarda and Suwała, 2016). The high overall smell, color, and general acceptance scores by probiotic potato chips covered with Tarkhineh especially turmeric Tarkhineh may be due to the extraordinary sensory properties of the fermentation process in Tarkhineh and the addition of spices such as turmeric to its constituents (Tabatabaei Yazdi et al., 1391). In contrast, the dramatic decline in sensory scores in non-probiotic potato chips may be related to the decrease in taste, odor, and color due to the accumulation of peroxides and free fatty acids in the absence of viable probiotic cells during four months of storage (Mostafa, 2020). Therefore, it is concluded that the production of probiotic potato chips covered with Tarkhineh is technically possible and cost-effective due to their high ability to maintain quality and sensory properties during shelf-life.

In conclusion, spraying of T34 probiotic bacterium using two Tarkhineh formulations on potato chips showed the best results including the highest viability during storage time and also acceptable increased sensory properties throughout four months period time at 4°C for T34 mixed with plain Tarkhineh (T34 + F1) formulation. T34 + F1 formulation preserved the probiotic cell viability at 1.4 × 10^7^ CFU g^–1^ and displayed high overall smell, color, and general acceptance scores during storage time. This study showed that traditional fermented dairy products such as Tarkhineh can be used as a blended matrix for probiotic formulation in potato chips. They offer additional benefits such as having prebiotic properties, as well as providing the minerals and vitamins needed.

## Data Availability Statement

The data presented in the study are deposited in the NCBI repositiry, accession numbers MW433680, MW433677, and MW433678.

## Ethics Statement

The studies involving “production of optimized potato using probiotic bacteria isolated from Kermanshah indigenous dairy products” were reviewed and approved by Mahmood Reza Moradi (Chairman of the Academic/Regional Ethics Committee in Biomedical Research) and Dr. Farbod Najafi (Secretary of the Academic/Regional Ethics Committee in Biomedical Research) in Kermanshah University of Medical Sciences.

## Author Contributions

AK: designing experiment. YN: writing. SH: data analysis. FG and DE: revising. BH: project administrator.

## Conflict of Interest

The authors declare that the research was conducted in the absence of any commercial or financial relationships that could be construed as a potential conflict of interest.

## Footnotes

1 http://blast.ncbi.nlm.nih.gov/Blast.cgi
